# Author’s reply to letter from the SIdP, Italian Society of Periodontology and Implantology on: Long-term efficacy of microbiology-driven periodontal laser-assisted therapy

**DOI:** 10.1007/s10096-016-2786-6

**Published:** 2016-10-13

**Authors:** F. S. Martelli

**Affiliations:** Microdentistry, Via dell’Ariento 4, 50123 Florence, Italy

Dear Editor,

We read the reply to our manuscript (“Long-term efficacy of microbiology-driven periodontal laser-assisted therapy”, *Eur J Clin Microbiol Infect Dis* 2016, 35 (3):423–431) and we would like to address those issues raised by the SIdP, Italian Society of Periodontology and Implantology.

## Diagnosis

We have to state that the diagnosis of periodontal disease in our manuscript was indeed based on current international standards as those proposed by the American Academy of Periodontology [1, 2]. In the American Academy of Periodontology Task Force Report on the Update to the 1999 Classification of Periodontal Diseases and Conditions, whose purpose was the development of a clinical interpretation of the *1999 Classification of Periodontal Diseases and Conditions,* it is stated that *“Formulation of a diagnosis of periodontitis is based on multiple clinical and radiographic parameters, all of which may not be required. In general, a patient would have periodontitis when one or more sites had inflammation (bleeding on probing [BOP]), radiographic bone loss, and increased probing depth or clinical attachment loss”.*

In the supplemental data of our manuscript, we state that “*Diagnosis of disease was made on the basis of dental clinical parameters, including periodontal probing depth (PPD), bleeding on probing (BOP), suppuration (PUS), and radiographic patterns of alveolar bone destruction*”. As is evident, these clinical parameters are in accordance with those referred in Table 1 of the American Academy of Periodontology Task Force Report, shown below, and meet the current international standards. Suppuration was used in our study as an extra parameter for determining the severity of periodontitis. As is known, suppuration has been used as a presumptive indicator of disease activity and is highly specific for disease progression [3].

**Table 1:** Guidelines for determining severity of periodontitis, as adapted from the American Academy of Periodontology Task Force Report [1]

	Slight (mild)	Moderate	Severe (advanced)
Probing depths	>3 & <5 mm	≥5 & <7 mm	≥7 mm
Bleeding on probing	Yes	Yes	Yes
Radiographic bone loss	Up to 15 % of root length or ≥2 mm & ≤3 mm	16 % to 30 % or >3 mm & ≤5 mm	>30 % or >5 mm
Clinical attachment loss	1 to 2 mm	3 to 4 mm	≥5 mm

With regard to the classification used, we included patients with periodontal pockets of 6 mm periodontal pocket depth or deeper, and we were not strictly restricted to patients of 7 mm periodontal pocket depth or deeper (as stated in Table 1 for patients with advanced chronic periodontitis).

In the introductory section of our manuscript, we stated that “*there is increasing evidence of a strong correlation between the presence of pathogenic bacteria in periodontal lesions and several systemic diseases. In particular, bacteremia and systemic endotoxin release can lead to the onset and progression of respiratory and cardiovascular diseases, rheumatoid arthritis and diabetes mellitus. Furthermore, oral bacteria can bypass the placenta filter, with a consequent increase of mediators responsible for infertility, preterm births and underweight newborns.”* As is shown, we mentioned in our manuscript the possible risk factors for periodontal disease, providing indicative references associated to this matter. However, in our study sample, no participants were diagnosed with any of the known risk factors that are detrimental for periodontal health. The patients selected had a clear medical history; hence, the study was limited only to smoking habits in relation to periodontal disease.

## Treatment

The SIdP, Italian Society of Periodontology and Implantology, state that “*in the last 15 years, RCTs from several independent universities and systematic reviews (SR) clearly showed a similar efficacy in applying classical therapy (mechanical SRP) or laser and no additional benefits in adding laser to mechanical treatment*”. However, from the scientific literature they cite, only one reference is associated specifically with Nd: YAG + SRP treatment [4]. Moreover, in this meta-analysis study [4], we find the following: “*All outcomes were evaluated from baseline to the end of follow-up. Significant differences in PD and GCF reduction were observed in favor of SRP + Nd: YAG; no significant differences were observed in CAL gain or PI change. The findings of this meta-analysis suggest that use of the Nd: YAG laser as an adjunctive therapy to conventional nonsurgical periodontal therapy could potentially provide additional benefits. However, all included studies were not at low risk of bias, and only three studies were included in the meta-analysis. As a result, the evidence is insufficient to support the effectiveness of adjunctive Nd: YAG to SRP. Future long-term well-designed parallel randomized clinical trials are required to assess the effectiveness of the adjunctive use of Nd: YAG laser. These trials should also include microbiological and adverse events analyses.*” As can be seen from this meta-analysis that examined specifically Nd: YAG type of laser, laser application adjunctively to SRP can be beneficial in reducing PPD. This observation was confirmed in our study, and microbiological analysis revealed that an enhanced microbiological environment of periodontal pockets could accompany this improved clinical parameter, as long as 24 months after initial therapy.

As this study is retrospective, we could not include control groups, because all our patients were treated according to the same protocol. In any case, rather than concluding that, as a consequence, “it is impossible for the reader to assess if final benefits are associated or not with laser application”, we would rather conclude that the results of the study warrant a prospective, randomized clinical trial, **including microbiological assessment**, comparing SRP alone with SRP + Nd: YAG laser application.

Furthermore, in their comments the authors fail to consider that dental roots and dentinal tubules (the reservoir of micro-organisms) do not have either blood vessels or a blood stream. Thus, antibiotics and antiseptics cannot reach the target pathogens, the infection endures, and the frequent, equally ineffective, use of antibacterial drugs can lead to resistance, an increasingly serious threat to global public health.

## Follow-up

Our definition of the final 2-year follow-up as a “long-term” observation, is based on previous scientific literature in which microbiology-specific periodontal studies define their measurements of less than 24-month follow-up of our study (even 8-month follow-up) as “long-term” [5–8]. In our study, whose purpose was to examine the effectiveness of our protocol in improving microbiological parameters of periodontal pockets, we observed that this persisted for even 24 months (thus, the “long-term” characterization is justified), and these long-term microbiological observations can be reflected in terms of clinical parameters (PPD, BOP, SUP).

## Statistical analysis

With regard to statistical analysis, the SIdP, Italian Society of Periodontology and Implantology, state that we used a *t*-test for statistical analysis, “which is not adequate in this type of study since multilevel analysis is generally used to assess the effect of prognostic factors on treatment outcomes”. Indeed, *t*-test is more adequate to our study, because it was not aimed at assessing the effect of prognostic factors on treatment outcome, but rather at assessing treatment outcome itself, and the outcome in different subpopulations of patients. The two cited papers instead have the specific aim of evaluating “Relative Contribution of Patient-, Tooth-, and Site-Associated Variability on the Clinical Outcomes of Subgingival Debridement”, and “Factors influencing the outcome of non-surgical periodontal treatment” respectively. Indeed, both works analyzed quite limited series of patients (94 and 41 respectively); such a limited sample size could not make it possible to answer the most critical question: “is the treatment equally effective in various subgroups of patients?”, because further subdividing the cases would ablate statistical power. Therefore, a multilevel regression analysis was instead used for an exploratory assessment of the contribution of various factors to the treatment outcome. In our work we were able to analyze a much larger set of cases, which made it possible to carry out our separate outcome analyses, and detect significant clinical and microbiological improvements in each of the subgroups analyzed. The fact that we could positively answer the most critical question (is the treatment effective?) by *t*-test in stratified subsets of patients, despite the known outcome variability, confirms the importance of analyzing large sets of cases, as we did. Further analyses exploring factors potentially influencing the outcome, and explaining its variability, are currently ongoing in our dataset, exploiting multilevel/multivariate analyses, but they are beyond the scope of the study under consideration.

## Ethics

Our study is retrospective, and as such, no formal consent is required (according to EJCMID instructions for authors that were written and formatted in accordance with the 1964 Declaration of Helsinki and its later amendments or comparable ethical standards). Furthermore, all patients participating in this study were anonymized, and informed consent was obtained from all subjects before their inclusion in this study, respecting the principle that all individuals have individual rights that are not to be infringed.

It is important to notice that in any case none of the components of the PERIOBLAST protocol (SRP, Nd: YAG laser treatment, microbiological assessment) can as of today be considered experimental, but rather commonly adopted options in standard practice. As technology advances, lasers have been widely used in all aspects of dentistry including periodontics, endodontics, orthodontics, and oral and maxillofacial surgery [9]. More specifically, among other types of lasers, Nd: YAG laser has been widely and safely used in various aspects of periodontology [10]. This retrospective study presents the results of a protocol that includes laser techniques applied in strict accordance with all precautions that should be taken to ensure their safe and effective operation, according to international standards [11]. Furthermore, this protocol includes slightly modified techniques used safely in the past and reported in the scientific literature, and it has been routinely and multicentrally applied by various clinicians in six different clinics, on their own responsibility. Finally, we have to emphasize that the basic purpose of this study is to retrospectively present details of this protocol applied, and its effectiveness in periodontal therapy.
